# Magnitude of Sexual and Reproductive Health Service Utilization Among Youths and Adolescents Living With Disability in Ethiopia: A Systematic Review and Meta‐Analysis

**DOI:** 10.1002/hsr2.70573

**Published:** 2025-03-20

**Authors:** Amare Mebrat Delie, Eneyew Talie Fenta, Molla Getie Mehari, Sileshi Berihun, Misganaw Guadie Tiruneh

**Affiliations:** ^1^ Department of Public Health College of Medicine and Health Sciences, Injibara University Injibara Ethiopia; ^2^ Departments of Medical Laboratory Science College of Medicine and Health Sciences, Injibara University Injibara Ethiopia; ^3^ Department of Health Systems and Policy Institute of Public Health, College of Medicine and Health Sciences, University of Gondar Gondar Ethiopia

**Keywords:** disability, Ethiopia, meta‐analysis, sexual and reproductive health, youths and adolescents

## Abstract

**Background and Aims:**

Sexual and reproductive health services are essential for the well‐being of youths and adolescents, including those living with disabilities. There was inconsistent evidence on the magnitude of sexual and reproductive health services utilization among youths and adolescents living with a disability. Therefore, this review aimed to assess the pooled magnitude of sexual and reproductive health service utilization among youths and adolescents living with a disability.

**Methods:**

Relevant databases including PubMed, Cochrane Library, AJOL, Semantic Scholar, Epistemonikos, Hinari, Google Scholar, and direct Google were used to search articles. This study included articles (both peer‐reviewed and preprints) in the English language from September 2013 to May 2, 2024. The pooled magnitude of sexual and reproductive health service utilization among youths and adolescents with a disability was estimated using a weighted DerSimonian‐laird random effect model. The *I*² statistics were used to ascertain the extent of heterogeneity. The funnel plot, Egger's regression, and Begg's test examined publication bias.

**Results:**

Based on this systematic review and meta‐analysis, the pooled magnitude of sexual and reproductive health service utilization among youths and adolescents with disability in Ethiopia was 29.11% (95% CI: 13.69, 44.53).

**Conclusions:**

The overall pooled magnitude of sexual and reproductive health service utilization among youths with disabilities was low in Ethiopia. The Ministry of Health and other collaborating organizations working on youth and adolescents with a disability should give special attention to enhancing the utilization of sexual and reproductive health services for youths and adolescents living with a disability. It is important to develop and create strategies that enable young people with disabilities to utilize sexual and reproductive health services, thereby protecting the rights and respecting the dignity of individuals with disabilities.

**Abbreviations**
CI: confidence interval
HIV: human immunodeficiency virus
JBI: Joanna Briggs Institute
PRISMA: Preferred Reporting Items for Systematic Reviews and Meta‐Analyses
SRH: sexual and reproductive health
SNNP: South Nations Nationalities and People
STI: sexually transmitted infection
WHO: World Health Organization

## 1 Introduction

Disability is defined by the World Health Organization (WHO) as any injury that limits an individual's ability to lead a normal life. Disabilities in any or all of the three domains of functioning, including impairment, activity limitation, and participation restriction, are referred to as disabilities [1]. According to a report from the WHO in 2011, approximately 15% of the global population has a disability [2]. In 2021, around 1.3 billion people, or 16% of the world's population, were estimated to be living with a disability [3].

About 33% of Ethiopia's population is made up of adolescents and young people, who have a significant impact on the social, political, and economic agendas of the nation. If investments are made to help them reach their health potential, they may also positively contribute to the country's growth [4]. Based on the 2015/16 Ethiopian household consumption expenditure survey, nearly 7.8 million people in Ethiopia are estimated to live with some form of disability, 9.3% of the country's total population. Approximately 30% of all individuals with disabilities are children adolescents and young people under 25 years of age [5].

Sexual and reproductive health (SRH) refers to physical and mental well‐being and encompasses the capacity to be free from unintended pregnancy, unsafe abortion, sexually transmitted infections, including HIV, and all types of sexual assault and coercion [6]. SRH problems are more common in adolescents because most people begin having sexual encounters at this time of their lives [6]. Even though young people's SRH is now widely acknowledged as a crucial public health issue [7], their ability to use SRH services has long been denied [8]. Managing sexual relationships can be complex and multifaceted, and this difficulty is increased when one has a disability [9, 10]. They often face barriers such as physical limitations, societal stigma, social isolation, and limited access to inclusive sexual education and healthcare services, all of which hinder their ability to navigate intimate relationships. Most of the time, the public thinks wrongly that young people with impairments are asexual [8]. The belief that individuals with disabilities are asexual arises from heteronormative views and insufficient education, fostering stigma and hindering the fulfillment of their sexual health needs [11]. Disabilities are diverse, affecting individuals in distinct ways, meaning their SRH needs vary. For instance, cisgender and transgender women, along with nonbinary individuals with disabilities, encounter unique challenges in accessing SRH services, highlighting the need for customized care [12]. Moreover, equating the experiences of cisgender and transgender individuals overlooks their distinct challenges, such as transgender individuals delaying or avoiding healthcare due to fear of discrimination [13]. Systematic review evidence from low‐ and middle‐income countries revealed that individuals with disabilities have a lower utilization rate of SRH services and a greater likelihood of unfavorable SRH outcomes [14].

The 2030 Agenda's Sustainable Development Goals (SDGs) for gender equality, good health, and well‐being all depend on universal access to SRH services [15]. For all groups to be able to make informed decisions, they must have access to SRH services [16, 17]. In May 2021, the 74th World Health Assembly endorsed an agreement stating that people with disabilities have the right to the best attainable standards of health, including SRH [18]. The Ethiopian National Adolescents and Youth Health Strategy (2021–2025) outlines priorities for improving the SRH of adolescents and youth, including adolescents and youth with disabilities in the country. Reaching these groups was given particular attention [4]. Ignoring the availability of SRH services for adolescents and youths has resulted in SRH problems. They might face a greater chance of becoming pregnant by accident, acquiring HIV or another STI, experiencing sexual manipulation or abuse, and being victims of violence [19]. Sexual manipulation involves using psychological pressure, deceit, or emotional tactics to control someone's sexual actions by exploiting vulnerabilities or trust without physical force [20]. Sexual abuse refers to any nonconsensual sexual act, often involving coercion, manipulation, or exploitation, violating trust and power dynamics, and leading to long‐term harm [21]. Sexual violence includes sexual acts, attempts, unwanted remarks or advances, or trafficking, perpetrated through coercion, intimidation, physical force, or threats, regardless of the relationship or setting [22, 23]. Healthy young people are very valuable and important. They can make a big difference in their families, communities, and country now and in the future. They can create positive change in society, instead of just relying on help from others [24].

Although Ethiopia has been working to make SRH services better for young people and has set up special services that are friendly for young people, child marriage, not being able to plan when to have children, and having children at a young age are common occurrences, especially in rural areas where more than 84% of people live [25]. In Ethiopia, a third of girls and young women between the ages of 15 and 19 have begun having children, with rural areas having a higher prevalence of early childbearing compared to urban areas [26]. Studies consistently showed that teenagers who begin sexual experimentation young and who lack access to or understanding of SRH services are less likely to utilize contraception. This, in turn, exposed them to undesirable outcomes like having numerous sexual partners and having unprotected intercourse, which can lead to an unwanted pregnancy, STIs, and depression [27, 28]. Every year, more than 2 million adolescents have unsafe abortions [29].

Given the large proportion of young people, including those with disabilities, and the ongoing poor SRH outcomes for these groups, it is imperative to understand SRH utilization to improve their health outcomes. This systematic review and meta‐analysis aimed to determine the pooled magnitude of SRH utilization in this population. By consolidating fragmented evidence, the review will offer a more accurate picture of service usage. Aggregating quantitative data from various studies in Ethiopia will provide pooled evidence on SRH utilization, offering reliable insights from diverse settings and informing the development of more inclusive and accessible public health policies. In addition to strengthening capacity among health policymakers and service providers and protecting the rights and dignity of people with disabilities, this research contributes to the development of normative tools, such as recommendations, to enhance disability inclusion in the health sector. To improve the use of SRH services by young people with disabilities, policymakers and the Ministry of Health must also implement specific interventions.

The review's research question was “What is the pooled prevalence of sexual and reproductive health service utilization among youths and adolescents living with disability in Ethiopia?”

## 2 Methods

### 2.1 Information Source and Searching Strategy

This systematic review and meta‐analysis were carried out in accordance with the Reporting Items for Systematic Reviews and Meta‐Analyses (PRISMA 2020) declaration [30]. The PRISMA checklist is available as Supporting Information (titled PRISMA checklist.docx). This review was registered in PROSPERO, with the registration number CRD42023441066. Relevant databases including PubMed, Cochrane Library, Semantic Scholar, Epistemonikos, Hinari, AJOL, Google Scholar, and direct Google were used to search articles. The study included articles (both peer‐reviewed and preprints) in English, published between September 2013 and May 2, 2024. The search was conducted using search terms related to the utilization of SRH services among youths with disabilities. We used Boolean operators “AND” and “OR” along with MeSH terms as follows: (((adolescent OR youth OR teenagers OR young) AND (sexual and reproductive health services OR sexual and reproductive health service utilization)) OR (youth‐friendly health service utilization OR youth‐friendly health services OR youth‐friendly health services uptake)) AND (disability OR impairment) AND (Ethiopia).

### 2.2 Eligibility Criteria

All English‐language, full‐text, original research articles and doctoral dissertations on cross‐sectional study design conducted in Ethiopia from September 2013 to May 2, 2024, that were published in peer‐reviewed journals or filed as completed dissertations that concerned SRH service utilization and among adolescents and youth living with disability in Ethiopia were included in this systematic review and meta‐analysis. We included studies related to SRH services utilization for youths and adolescents with disability aged 10–24 years. In contrast, editorials, reports, and studies that lack prevalence or proportion of SRH service utilization were eliminated. Studies on SRH service utilization that reported only descriptive survey results, without statistical analysis accounting for probability sampling and generalizability, were also excluded.

For this review, the following operational definitions were used:

SRH service utilization was defined as ever utilization of any one of the following SRH services: SRH information, education and counseling, contraceptive service, pregnancy test and care, voluntary counseling and testing (VCT), STI screening, diagnosis, and management services, and safe abortion care [31]. Adolescents are individuals between the ages of 10 and 19, whereas youth are those between the ages of 15 and 24, according to the World Health Organization (WHO) [32].

Disability: Adolescents or youths with physical, visual, hearing, or mental impairments were classified as disabled.

### 2.3 Data Extraction

Articles from relevant databases were exported into Endnote Library reference manager software version 20. The articles were then gathered into a single folder so that the aforementioned software could identify and eliminate duplicates. After initial screening, three reviewers (M.G.T., S.B., and E.T.F.) downloaded abstracts to assess them for inclusion. If reviewers disagreed on whether a search result was relevant to the study, it was retained for retrieval. The relevance of the items was then assessed using the title, topic, objectives, and methods given in the abstracts. Abstracts were also evaluated for consistency with the inclusion criteria. At this stage, articles considered that did not meet the criteria or were out of the scope of the study were excluded, and the full text of the remains was downloaded for a detailed review. If there was uncertainty regarding the relevance of an abstract or if reviewers disagreed on whether it met the inclusion criteria, it was selected for retrieval.

### 2.4 Data Quality Assessment

Three reviewers (A.M.D., E.T.F., and M.G.T.) then assessed the quality of potentially eligible articles using the Joanna Briggs Institute (JBI) critical appraisal checklist available at https://jbi.global/critical-appraisal-tools. Using the tool as a protocol, the reviewers used the blinded review approach to evaluate the quality of the original articles. The average of three independent reviewers' scores was used to determine whether or not the articles should be included. Those studies with scores of 5 or more in JBI criteria were considered to have good quality and were included in the review [33]. In this review, there were no discrepancies between the authors in the quality assessment, so there was no need to involve a fourth reviewer to resolve any disagreements. All the necessary data had already been accessed by downloading the full texts of the articles included in the review. Finally, extracted data from endnote were exported into Microsoft Excel version 2021 based on a standardized data extraction checklist. The name of the author, study setting, study design, publication, sample size, response rate, and proportion of SRH service utilization were included in our data extraction checklist (Table 1).

**Table 1:** Methodological quality assessment of included studies using the JBI critical appraisal checklist.

Study	Was the sample frame appropriate to address the target Population?	Were study participants sampled appropriately?	Was the sample size adequate?	Were the study subjects and the setting described in detail?	Was the data analysis conducted with sufficient coverage of the identified sample?	Were valid methods used for the identification of the condition?	Was the condition measured in a standard, reliable way for all participants?	Was there an appropriate statistical analysis?	Was the response rate adequate, and if not, was the low response rate managed appropriately?	Total
Diribsa et al.	Yes	Yes	Yes	Yes	Yes	Yes	No	Yes	Yes	7
Shehu et al.	Yes	Yes	Yes	Yes	Yes	Yes	Yes	Yes	Yes	9
Mesfin	Yes	Yes	Yes	No	Yes	Yes	Yes	Yes	Yes	7
Kassa et al.	Yes	Yes	Yes	Yes	Yes	Yes	Yes	Yes	Yes	9

### 2.5 Data Analysis

Data were extracted from eligible studies using Microsoft Excel Version 2021 and exported to STATA version 17 software for statistical data analysis. The articles were summarized with tables and forest plots. The standard error and 95% confidence interval for the proportion of SRH service utilization were calculated for those studies in which estimates of standard error and 95% confidence interval for the proportion of SRH service utilization were not found in the full text of their article. The meta‐analysis was performed to estimate the pooled proportion of SRH service utilization among youths and adolescents with disabilities, using a 5% level of significance and two‐sided tests. Statistical heterogeneity was evaluated subjectively using forest plots and objectively with the Cochrane *Q*‐test and *I*² statistics [34]. Accordingly, the analysis revealed a considerable heterogeneity (*I*² = 98.21%, *Q* = 213.41, *p* < 0.001). An *I*² value of 98.21% indicates that 98.21% of the variability in the pooled estimate is due to differences between studies rather than random sampling error. This considerable heterogeneity suggests substantial variations in study characteristics, such as sample size, population demographics, or measurement methods. The *Q*‐statistic of 213.41 with *p* < 0.001 further confirmed the presence of statistically significant heterogeneity, indicating that the observed variability exceeds what would be expected by chance alone. This result underscores the need for a model that accounts for such variability. Therefore, a random‐effects model was applied to generate the pooled estimate of SRH service utilization among disabled youths, as it accounts for both within‐ and between‐study variability. The presence of publication bias was checked by subjectively using a funnel plot asymmetry and objectively using Egger's and Begg's statistical tests [35]. Based on these assessments, we concluded that publication bias does not significantly affect the findings of our review on the utilization of SRH services among adolescents and youths with disabilities.

## 3 Results

**Figure 1:**
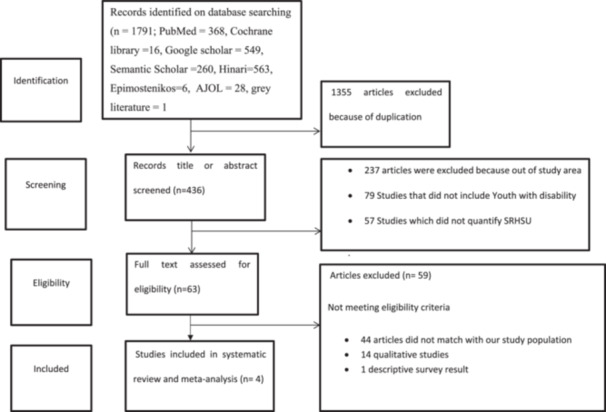
PRISMA flow diagram of study selection on sexual and reproductive health service utilization among youths and adolescents living with disability in Ethiopia.

After searching in different databases, we found 1791 articles: 368 articles in PubMed, 549 articles in Google Scholar, 16 articles in Cochrane Library, 260 articles in Semantic Scholar, 563 articles in Hinari, 6 articles in Epimostenikos, 28 articles in AJOL, and 1 article in gray literature. Then 436 records were examined by title and abstract after duplicate records were eliminated. For the full‐text review, 63 papers were included. Finally, four studies were eligible for quantitative synthesis (Figure 1).

### 3.1 Characteristics of the Included Studies

In the meta‐analysis, four studies were included. These studies were conducted in Amhara [36], Southern Nations, Nationalities and Peoples (SNNP) [37], Oromia [38], and Addis Ababa city [39] of Ethiopia. All the studies included in the review used a cross‐sectional design. Of all the studies, one study [36] utilized a community‐based cross‐sectional design, whereas the remaining three studies [37, 38, 39] used an institution‐based cross‐sectional study design. Among the institution‐based studies, two [37, 38] were conducted in school settings, whereas one study [39] was conducted in organizations supporting individuals with disabilities, such as associations for the physically handicapped, blind, deaf, deaf‐blind, leprosy patients, and the mentally handicapped. The total sample size for this review was 1542 [36, 37, 38, 39]. The highest utilization of SRH service was 42% in a community‐based cross‐sectional study conducted by Shehu et al. [36]. This study was conducted among youths aged 15–24 with disabilities in Dessie city. In contrast, the lowest SRH service utilization was 8.4%, as Diribsa et al. [38] reported in a study of primary and secondary school adolescents aged 10–19 years with disabilities in the Jimma zone. Kassa et al. [39] found SRH service utilization of 26.1% among young people aged 10–24 years with disabilities in Addis Ababa. The study populations with Diribsa et al. [38] and Mesfin [37] were taken from in‐school students, whereas Shehu et al. [36] and Kassa et al. [39] sampled from the community. All studies reviewed examined the utilization of SRHS among young people with disabilities (Table 2) [36, 37, 38, 39].

**Table 2:** Summary of characteristics of included articles for a systematic review and meta‐analysis of SRH service utilization among youths and adolescents living with disability in Ethiopia.

First author	Publication year	Region	Study design	Target gender	Study area	Study population	Sample size calculated	Sample size used in male	Sample size used in female	Total sample size used	Response rate (%)	SRH service utilization (%)
Diribsa et al. [38]	2022	Oromia	Institutional‐based cross‐sectional	Both	Jimma zone	Adolescents with disabilities aged 10–19 years	466	258	196	454	97.4	8.4
Shehu et al. [36]	2021	Amhara region	Community‐based, cross‐sectional study	Both	Desie city	Youths with disabilities aged 15–24 years	423	196	190	386	91	42
Mesfin [37]	2022	SNNP	Institutional‐based cross‐sectional	Both	Arba Minch town	Adolescents with disability aged 15–19 years	227	110	101	211	92.95	40.52
Kassa et al. [39]	2023	Addis Ababa	Institutional‐based cross‐sectional	Both	Addis Ababa town	Young with disability aged 10–24 years	426	274	152	426	100	26.1

Abbreviations: SNNP, South Nation Nationalities Ethiopia; SRH, sexual and reproductive health.

### 3.2 Magnitude of SRH Service Utilization Among Youths and Adolescents Living With Disability

**Figure 2:**
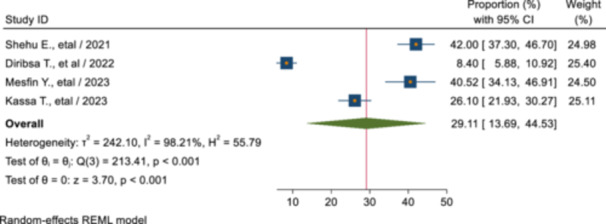
Pooled magnitude of sexual and reproductive health service utilization among youths and adolescents with disability in Ethiopia.

The pooled magnitude of SRH service utilization among youths and adolescents living with disability in Ethiopia was 29.11% (95% CI: 13.69, 44.53) (Figure 2).

### 3.3 Publication Bias Assessment

**Figure 3:**
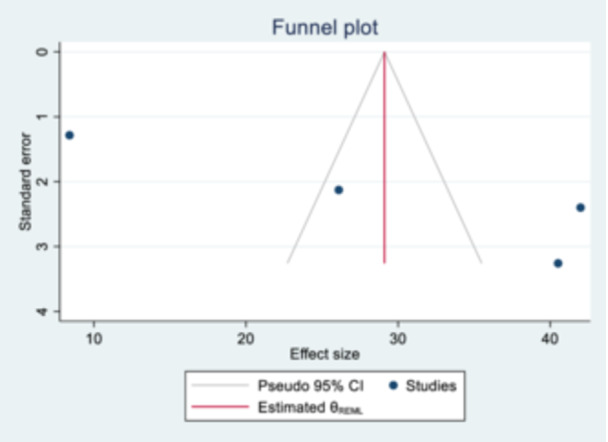
Publication bias assessment using funnel plot for sexual and reproductive health service utilization among youths and adolescents with disability in Ethiopia.

The assessment of publication bias was conducted using both a funnel plot for a subjective analysis and Egger's and Begg's tests for an objective evaluation. In this study, a funnel plot showed a symmetrical distribution (Figure 3). Eggers's test detected publication bias at a significance level of 0.05, with *p* = 0.006. In contrast, Begg's test found no evidence of publication bias at the same significance level, with *p* = 0.31. We also used a no‐parametric trim and fill study in the random effects model to reduce the impact of publication bias. After conducting Duval and Tweedie's nonparametric trim and fill analysis, the pooled proportion estimates between the original model and the trim and fill model did not change substantially and did not account for additional studies. Therefore, we conclude that publication bias does not impact the final results of our review on the utilization of SRH services by adolescents and youths with disabilities.

### 3.4 Sensitivity Analysis

**Figure 4:**
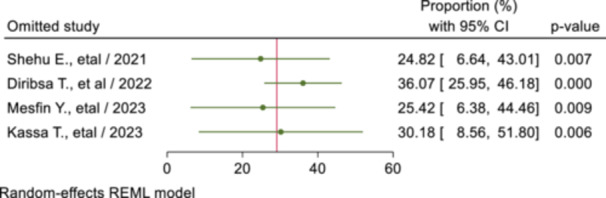
Sensitivity analysis for the pooled proportion of sexual and reproductive health service utilization among youths with disabilities in Ethiopia.

Through a step‐by‐step elimination of each study, a meta‐leave‐out‐one sensitivity analysis was conducted to quantify the impact of each study on the pooled magnitude of SRH utilization. The outcome demonstrated that no studies were found to be outside the pooled proportion of SRH service utilization's confidence interval. Consequently, by removing one study from the meta‐analysis, it was demonstrated that all studies had an almost identical influence on the overall pooled proportion of SRH service utilization (Figure 4).

## 4 Discussion

People with disabilities have SRH needs that are equivalent to those of others [40]. SRH services are essential for safe and healthy relationships as well as protection against HIV and other STIs. However, individuals with disabilities frequently encounter challenges in accessing these services. The pooled prevalence of SRH service utilization among adolescents and youths with disabilities in Ethiopia was 29.11%, reflecting an alarmingly low rate of utilization. This finding underscores significant systemic inequities and barriers faced by youths and adolescents with disability in accessing essential SRH services. Global reports indicate that individuals with disabilities are two to three times more likely to experience unmet healthcare needs, including SRH services [41]. It highlights an urgent need for targeted interventions and inclusive policies to address these disparities and ensure equitable access to SRH services for this vulnerable population. This result is lower than the findings from Ghana, 65.4% [42]. This finding is also not significantly varied with a study in Ethiopia, 42.73% [43], and Uganda, 42.0% [44] which were conducted among youths aged 15–24 years. However, this result is higher than the findings from Nepal, 9.2% [45]. This discrepancy might be due to variations in the country's healthcare infrastructure and accessibility, inclusive policies, or perhaps more comprehensive SRH programs tailored to the needs of individuals with disabilities. The other reason for this difference might be due to the difference in their sociodemographic characteristics, cultural attitudes toward disability, and the overall accessibility of SRH services. This discrepancy could be attributed to differences in healthcare infrastructure, accessibility, and the presence of inclusive policies or comprehensive SRH programs specifically designed to address the needs of individuals with disabilities. Additionally, variations in socio‐demographic characteristics, cultural perceptions of disability, and the overall accessibility of SRH services may also contribute to these differences.

## 5 Limitation

Although we followed PRISMA guidelines for conducting this review, the number of studies incorporated in the final meta‐analysis was few, which may reduce the statistical power and generalizability of the findings. The other possible drawback of the current systematic review could be that it only included full‐text publications written in the English language, which means important works that may have been written in other languages on the subject were missed, potentially introducing language bias.

## 6 Conclusion

The overall magnitude of SRH service utilization among youths and adolescents with disabilities was found to be low in Ethiopia. This meta‐analysis highlights Ethiopia's current position as a starting point for improving the lives of youths and adolescents with disabilities. However, fulfilling the mandate of Ethiopia's National Adolescent and Youth Health Strategy will require disability‐inclusive approaches and targeted interventions to address barriers to service access and uptake. Collaborations with organizations such as the Ethiopian Center for Disability and Development, the Family Guidance Association of Ethiopia, and the Federation of Ethiopian Associations of Persons with Disabilities remain crucial to building healthcare provider capacity, enhancing accessibility, and promoting community‐based awareness programs. Additionally, nongovernmental organizations and international partners such as the United Nations Population Fund can play a pivotal role in supporting these efforts through technical and financial assistance. These actions will help to scale up inclusive SRH services, enabling Ethiopia to better serve the needs of youths and adolescents with disabilities. These targeted interventions can also significantly enhance SRH service utilization among youths and adolescents with disabilities in Ethiopia. Future efforts should focus on identifying and addressing barriers to SRH service utilization to ensure equitable access for all.

## Author Contributions

A.M.D., S.B., and M.G.T. developed the protocol and were involved in the design, conceptualization, data extraction, statistical analysis, and the development of initial drafts of the manuscript. A.M.D., M.G.T., S.B., M.G.M., and E.T.F. were involved in data extraction and quality assessment. All authors have read and approved the final version of the manuscript. The corresponding author had full access to all of the data in this study and took complete responsibility for the integrity of the data and the accuracy of the data analysis.

## Ethics Statement

The authors have nothing to report.

## Consent

The authors have nothing to report.

## Conflicts of Interest

The authors declare no conflicts of interest.

### 1 Transparency Statement

The corresponding author, Amare Mebrat Delie affirms that this manuscript is an honest, accurate, and transparent account of the study being reported; that no important aspects of the study have been omitted; and that any discrepancies from the study as planned have been explained.

## Supporting information

Supporting information.

## Data Availability

The authors confirmed that the data supporting the findings of this study are available within the article and its Supporting Information.
